# Evaluating the Applicability of the TOMCAST Model for the Control of Potato Early Blight in China

**DOI:** 10.3390/plants12081634

**Published:** 2023-04-12

**Authors:** Qing Li, Xueyan Zhang, Xin Ma, Hailong Li

**Affiliations:** 1 Institute of Geographical Science and Natural Resources Research, Chinese Academy of Sciences, Beijing 100101, China; 2 Institute of the Environment and Sustainable Development in Agriculture, Chinese Academy of Agriculture Sciences, Beijing 100081, China; maxin02@caas.cn; 3 Xuechuan Agricultural Group Co., Ltd., Zhangjiakou 076481, China

**Keywords:** TOMCAST, potato early blight, fungicide application, potato yield, quality, China

## Abstract

To explore the applicability of different fungicide application schemes to control potato early blight (mainly caused by *Alternaria solani*) in China, field trials were conducted from 2020 to 2022, combining different fungicides with the tomato forecaster (TOMCAST) model and using weather variables to adjust the minimum temperature of TOMCAST to 7 °C. To effectively manage potato early blight, the TOMCAST model combines relative humidity (>88%) and air temperature to calculate daily severity values (DSVs). The application of fungicides (fungicide schedule) is as follows: untreated; two standard treatments, Amimiaoshou SC and Xishi SC, applied at the first appearance of disease symptoms; and two different TOMCAST treatments, in which fungicides are applied when the physiological days add up to 300 days and DSVs add up to 15. This study quantifies the intensity of early blight as the area under the disease progression curve and the final disease severity. Additionally, a progress curve for early blight is constructed to compare the development of early blight in different years and treatments. The TOMCAST-15 model reduces the number of fungicide applications in addition to significantly suppressing the development of early blight. Furthermore, fungicide application significantly increases the dry matter and starch contents of potatoes, and TOMCAST-15 × Amimiaoshou SC has similar enhancement effects on dry matter, protein, reducing sugar, and starch contents compared with Amomiaohou SC and Xishi SC. As a result, TOMCAST × Amimiaoshou SC may be an effective alternative to the standard treatment and have good applicability in China.

## 1. Introduction

Potato (*Solanum tuberosum L*.) is the world’s largest non-cereal crop and the fourth most important food crop in the world, after rice, wheat, and corn [1]. China is the world’s largest producer of potatoes. In 2019, China’s potato planting area reached 4.67 million hm^−2^ [2]. However, the average potato yield in the past 10 years was 16.4 t hm^−2^ [3], which is significantly lower than the European and world averages. Among them, diseases and insect pests are key factors that restrict the increase in the average yield of potatoes in China.

Early blight is one of the most common foliar diseases in potato-growing regions worldwide [4]. It is a fungal disease caused by *Alternaria solani* and characterized by irregular dark concentric ring-shaped lesions, particularly on potato leaves, petioles, and tubers [5]. If no effective measures are taken to prevent and control it, early blight disease will intensify rapidly, resulting in a loss of approximately 20% of a potato yield [6].

Late blight, mainly caused by *Phytophthora infestans* (Mont.) de Bary, has always been the most important foliar disease of concern to potato growers in China. In recent years, the rapid growth of early blight has become an unavoidable issue. The high temperature and alternating rainfall in summer have contributed to the increase in early blight; simultaneously, fungicides effective against both late and early blight have transformed into fungicides that are only effective against late blight [7]. As a result, different fungicides are required to control the early blight. Early blight can occur anywhere potatoes are grown, but the severity varies from year to year, depending primarily on weather conditions (mainly temperature and humidity), potato variety, and other factors. The application of fungicides is the main method to control early blight because of the lack of resistant potato cultivars. The accurate and timely application of fungicides for efficient crop management can inhibit the formation and development of early blight. In most potato-growing areas in China, growers usually start to apply fungicides in the early stage of early blight based on previous experience, regardless of climatic conditions, environment, and other factors [8,9]. It is crucial to regulate fungicide treatments to limit overapplication for the prevention of early blight because of the concerns such as the overuse of fungicides that cause environmental contamination and pose a risk to human health. One method to regulate their application is to use predictive models or rules to determine when fungicides are required and manage them [8,10]. The use of a decision support system (DSS) to reduce crop losses can rationalize their use [4,11]. Simultaneously, using DSS can reduce the number of fungicide sprays while providing better disease control [12].

Tomato forecaster (TOMCAST), a DSS for early blight, was developed based on disease charts from the forecast of the *A. solani* tomato (FAST) program cross-referenced with temperature and leaf wetness [13]. Combined with leaf wet hours and the average air temperature, the increased daily severity value (DSV) under weather changes was estimated. This approach was originally used in the FAST program to calculate DSV [14] and accumulate severity values up to 10, 15, 20, 25, etc. It was subsequently applied in the field management of crops such as asparagus [15], carrots [16], and potatoes [17]. Studies have shown that 15 DSV is the optimal threshold for field management of the TOMCAST model and has the best control effect on the early blight [10,12].

Currently, to our knowledge, there is no potato early blight prediction system based on climatic conditions in China to support the field management of fungicide applications. Therefore, our goal was to (1) evaluate the control effect of different fungicides and (2) explore whether TOMCAST 15 DSV is suitable for potato production in China.

## 2. Results

### 2.1. Weather Conditions during the Trials

**Figure 1:**
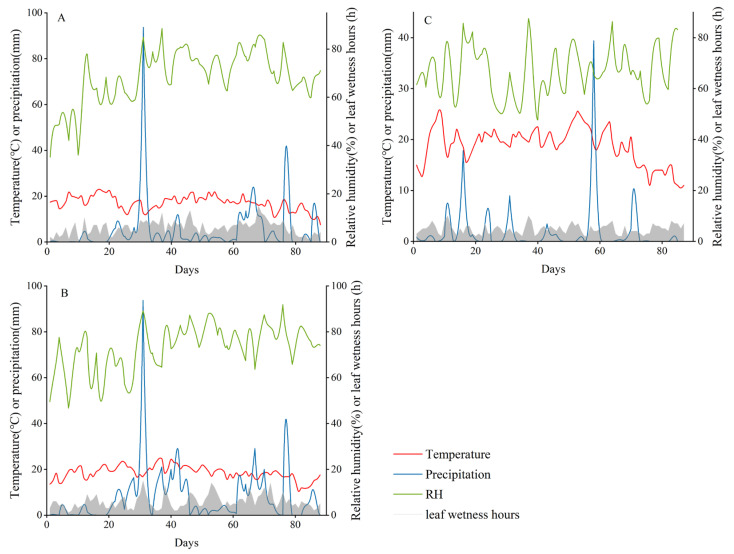
Leaf wetness hours, relative humidity, air temperature, and precipitation were measured daily by a weather station in the experiments conducted from June to August of 2020 (**A**), 2021 (**B**), and 2022 (**C**).

The weather conditions during the trials from 2020 to 2022 are summarized in Figure 1. The comparison revealed that the average temperature, relative humidity (RH), and cumulative monthly rainfall in June, July, and August in 2020 and 2021 did not differ significantly. The average values of the daily temperature in June, July, and August 2020 were 19.30, 18.64, and 18.37 °C, respectively. The average RH in June, July, and August was 58.25, 76.84, and 77.30%, respectively. The total precipitation in June, July, and August was 87.78, 254.59, and 266.36 mm, respectively (Figure 1A). In 2021, the average values of daily temperature during the trial in June, July, and August were 18.78, 20.40, and 16.38 °C, respectively. The average RH was 59.69, 75.32, and 74.60% in June, July, and August, respectively. Total precipitation was 74.93, 348.26, and 276.87 mm in June, July, and August, respectively (Figure 1B). However, the temperature in 2022 increased compared with that in both previous years. There was a significant decrease in precipitation. The daily temperature ranged from 2.3 to 33.2 °C; the mean RH was 65.68, 58.31, and 66.49% in June, July, and August, respectively. Total precipitation was 43.27, 74.53, and 89.32 mm in June, July, and August, respectively (Figure 1C). In addition, the number of leaf-wetting hours in 2022 was marginally lower than those in the previous two years.

In general, the meteorological conditions in these three years were favorable for the development of early blight. The meteorological conditions were generally similar in these three years, except for in 2022, when there was less precipitation.

### 2.2. Fungicide Applications

The fungicide applications during the field experiment are presented in Table 1 (2020), Table 2 (2021), and Table 3 (2022). Over the three years, TOMCAST-15a and TOMCAST-15b resulted in the lowest number of fungicide applications. The TOMCAST threshold (15) resulted in the application of four fungicides from 2020 to 2022 (Table 1, Table 2 and Table 3)—all less than the standard treatment. The TOMCAST treatments recommended the first fungicide application nine days earlier than the standard treatment, 14 days in 2021, and 21 days in 2022.

### 2.3. Progression of Potato Early Blight from 2020 to 2022

**Figure 2:**
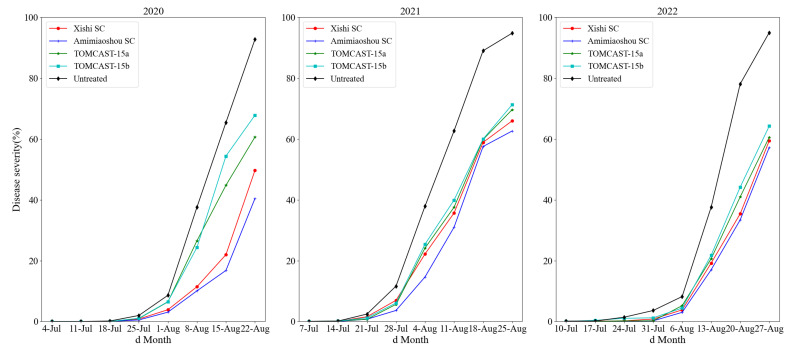
The disease progression of early blight as a function of the different fungicide treatment schedules from 2020 to 2022.

Potato early blight was the dominant disease in these three years. Its first symptoms were discovered on 18 July 2020, 21 July 2021, and 31 July 2022. During the three-year field experiments, potato early blight developed rapidly in untreated field plots, and the disease severity of early blight reached more than 90% in the week before potato harvest (Figure 2).

Application of the fungicide inhibited the development of early blight compared with the untreated plots, according to a visual analysis of the disease progression curve (Figure 2). In 2020, the development of early blight in the standardized Amimiaoshou-SC- and Xishi-SC-treated plots was significantly slower than in those sprayed with TOMCAST-15. However, in 2021 and 2022, the changing trend of early blight between the standardized treatment of Amimiaoshou SC and Xishi SC and the spraying treatment of TOMCAST-15 is not apparent. Overall, the results of three consecutive years of field trials showed that the plots treated with Amimiaoshou SC and Xishi SC had the slowest development of potato early blight, followed by the plots sprayed with TOMCAST-15 × Amimiaoshou SC and TOMCAST-15 × Xishi SC (Figure 2).

### 2.4. The Effect of Different Treatments on the Final Severity and Area under the Disease Progress Curve (AUDPC) of Early Blight

**Figure 3:**
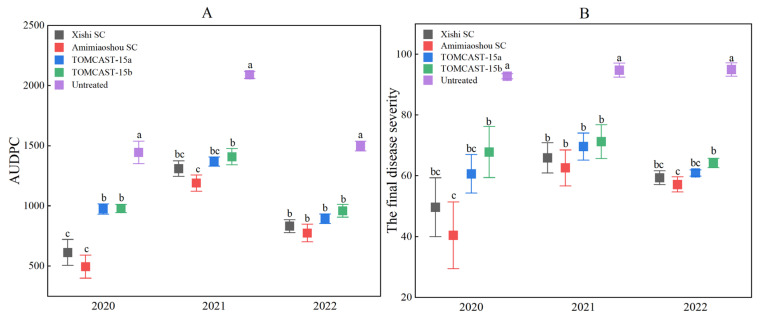
Comparison of area under the disease progression curve (AUDPC) (**A**) and the final disease severity (**B**) from 2020 to 2022. Each point represents the mean of five replicates, and the error bars associated with each point represent the standard error. The same letter on each point indicates no significant difference between different treatments in the same year, and vice versa, according to LSD’s honestly significant difference (*p* < 0.05).

During these three years, there was a significant difference (*p* < 0.01) between the four applied treatments for early blight and the control group. The application of fungicides achieved good control of early blight (Figure 2). Compared with other treatments, the Amimiaoshou SC standardized application scheme had the best inhibitory effect on early blight. The final disease index was the lowest (Figure 3B), followed by Xishi SC. As shown in Figure 3B, the trends in final disease severity in 2020 and 2022 were relatively consistent, with the lowest final disease severity values for Amimiaoshou SC, followed by Xishi SC, then TOMCAST-15a and TOMCAST-15b. However, there were no statistically significant differences in final severity values among the Amimiaoshou SC, Xishi SC, and TOMCAST-15a treatments, nor among the Xishi SC, TOMCAST-15a, and TOMCAST-15b treatments. In 2021, there was no significant difference in the final severity values among the four treatments: Amimiaoshou SC, Xishi SC, TOMCAST-15a, and TOMCAST-15b. 

The AUDPC for each subplot was used as a critical measure of resistance [18]. In this three-year field trial, the different fungicide schedules had significant effects on the final severity and AUDPC of potato early blight (*p* < 0.01) (Figure 3). In 2020, compared with other fungicide treatments, the lowest AUDPC was observed for the standard application of Amimiaoshou SC. There was no significant difference between the two fungicide schemes, TOMCAST-15a and TOMCAST-15b. However, both these treatments had higher AUDPC values than Amimiaoshou SC and Xishi SC. The AUDPC results for 2021 were similar to those in 2020, with Amimiaoshou SC having the lowest AUDPC, followed by Xishi SC, then TOMCAST-15a and TOMCAST-15b, but there was no significant difference among the three treatments, Amimiaoshou SC, Xishi SC, and TOMCAST-15a, nor among treatments with Xishi SC, TOMCAST-15a, and TOMCAST-15b (Figure 3A). In 2022, the AUDPC results were different from those of the previous two years, with no differences among Amimiaoshou SC, Xishi SC, TOMCAST-15a, and TOMCAST-15b.

### 2.5. Tuber Yield and Quality

The average tuber yields from the experiments conducted from 2020 to 2022 are listed in Table 4. There was no significant effect of the different fungicide treatments on potato tuber yield in 2020 (*p*_2020_ = 0.08), 2021 (*p*_2021_ = 0.36), and 2022 (*p*_2022_ = 0.06). As a result, no further analysis was performed.

The quality of potatoes primarily depends on the composition and content of tubers, among which nutrients such as starch, protein, and reducing sugars are the primary indicators for measuring the quality of potato tubers [18]. Compared with tuber yield, fungicide application had a significant effect (*p* < 0.05) on potato quality indicators, such as dry matter, starch, protein, and reducing sugar contents. From 2020 to 2022, the dry matter, starch, protein, and reducing sugar contents of potatoes in the application program were higher than those in the untreated application.

**Figure 4:**
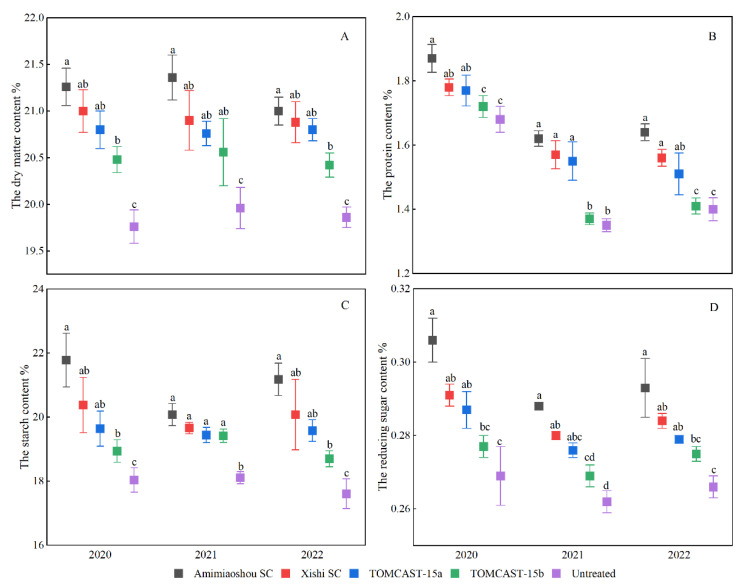
Comparison of potato dry matter (**A**), starch (**C**), protein (**B**), and reducing sugar (**D**) contents from 2020 to 2022. Each point represents the mean of five replicates, and error bars associated with each point represent the standard error. The same letter on each point indicates no significant difference between different treatments in the same year and vice versa, according to LSD’s honestly significant difference (*p* < 0.05).

A comparison of the four different fungicide application regimens revealed that the dry matter, starch, protein, and reducing sugar contents were the highest for the Amimiaoshou SC application, but the differences were not significant relative to the Xishi SC and TOMCAST-15a (Figure 4). The dry matter, starch, protein, and reducing sugar contents of TOMCAST-15b were significantly lower than those of Amimiaoshou SC, Xishi SC, and TOMCAST-15a from 2020 to 2022. Compared with TOMCAST-15a, TOMCAST-15b had no significant difference in dry matter, starch, and reducing sugar contents, but there were significant differences in protein content. Additionally, the dry matter and starch contents of the TOMCAST-15b treatment from 2020 to 2022 were significantly different when compared with the untreated samples (Figure 4A,C). However, there was no difference in protein content (Figure 4B) and no significant differences in reducing sugar content (Figure 4D).

## 3. Discussion

The severity and development area of potato early blight have changed in recent years because of changes in climatic factors. If preventive measures are not taken, leaves will senesce earlier, and growers will be at risk of reduced yields, seed potato quality, and economic profitability. Currently, most potato growers in China opt to spray fungicides frequently based on experience to prevent early blight when the risk period comes. However, starting blind applications when they see early blight based on experience can contaminate the soil and environment, which is not conducive to environmental sustainability.

Adopting a disease forecasting system is an effective measure for optimizing the application of fungicides and provides an integrated disease management program for potato production. Currently, the main forecast systems used in agricultural disease control are the PLANT-Plus [11] and the TOMCAST models [13,15,19]. PLANT-Plus is an application software package. It is a computer program developed to simulate and calculate disease severity values based on potato early blight. This approach also reduces the number and frequency of fungicide applications compared with traditional weekly fungicide applications; however, operating it requires a certain level of computer skills and increases input costs for growers. TOMCAST is a simple meteorological model. It achieved good results in control experiments on potato early blight in Denmark and Spain. It reduces the number of sprays but also improves the control effect of early blight [10,20]. However, only a few studies in China have shown that effective control similar to conventional experience can be achieved when fungicides are applied from the first symptoms of early blight [3], and a DSS incorporating weather conditions is lacking. Therefore, the issue of comparing existing fungicide application programs with the TOMCAST model to establish a field management program and exploring whether it can achieve good control effects in China have not yet been clarified.

The weather conditions in the past three years were all favorable for the occurrence of early blight, but the onset time and trend of early blight in the untreated field plots were not consistent. One possible explanation is that weather conditions, such as temperature and precipitation, vary from year to year (Figure 1). Simultaneously, the plots we tested may have been affected by diseases from neighboring experimental plots. The degree of early blight development in adjacent plots in 2020 was earlier than that in 2021 and 2022 (Table 1, Table 2 and Table 3); therefore, the airborne conidia of *A. solani* may have appeared earlier in the field test plots in 2020. At the same time, as shown in Figure 2, from 2020 to 2022, Amimiaoshou SC has the best control effect, followed by Xishi SC, which is related to the main components of the pesticide itself. The main component of Amimiaoshou SC is difenoconazole-azoxystrobin, and that of Xishi SC is chlorothalonil; this conclusion is similar to that of Dorman et al. [21].

Using the TOMCAST model, fungicides were applied at critical times when the weather was favorable for the development of early blight. Compared with the standard treatment, fewer fungicides were used to control early blight (Table 1, Table 2 and Table 3). Some studies have also suggested using predictive models to target the use of fungicides at the key time of the season when the weather is favorable for the onset of early blight to improve the efficiency of fungicides in controlling early blight [4]. The TOMCAST 15 DSV program used in this study reduced the spraying of leaves by two times compared with the regular weekly interval spraying treatment of Amimiaoshou SC and XishiSC (Table 1, Table 2 and Table 3). Among them, TOMCAST-15a and TOMCAST-15b had significantly different inhibitory effects on the disease compared with no treatment in terms of AUDPC and severity of the final disease. However, compared with the Amimiaoshou SC and XishiSC treatments, TOMCAST-15a was better than TOMCAST-15b at controlling early blight. Therefore, the inhibitory effects of the two TOMCAST application regimens on early blight were as follows: TOMCAST 15 DSV× Amimiaoshou SC >TOMCAST 15 DSV× XishiSC.

There were no differences in tuber yield among the different fungicides and application schemes (Table 4). This should be due to measuring tuber yield by fresh weight without correcting for water. This result is consistent with the results determined by Abuley and Nielsen [10]. Additionally, as shown in Figure 4, there are differences in the effects of different application schemes on the dry matter, starch, protein, and reducing sugar contents of potatoes. Furthermore, compared with the untreated scheme, the other four application schemes can significantly increase the dry matter and starch contents of potatoes; this phenomenon occurs because starch accounts for 65–80% of the dry matter content of potato tubers [22], providing support for a strong correlation between dry matter and starch contents [23]. There is no significant difference in protein and reducing sugar contents among the three treatments of Amimiaoshou SC, XishiSC, and TOMCAST-15a, as well as between TOMCAST-15b and the untreated schemes. This result should come about because the final disease severity of early blight in TOMCAST-15b treatment is higher, which affects the absorption and accumulation of nitrogen content in potato fertilization [24], resulting in low protein and reducing sugar content. This finding is consistent with the conclusions of previous studies that as the severity of the disease increases both protein content and reducing sugar contents decrease significantly [25].

## 4. Materials and Methods

### 4.1. Experimental Site and Design

During the 2020, 2021, and 2022 potato growing seasons, the field experiments were conducted in a commercial potato field located in the Xuechuan Potato Plantation, Zhangjiakou (113° 50′ E, 39° 30′ N). The soil type at the experimental site was sandy loam [26]. The experimental sites were preceded by a 2-year potato-free period from 2017 to 2019, during which naked oats were grown in the field.

**Figure 5:**
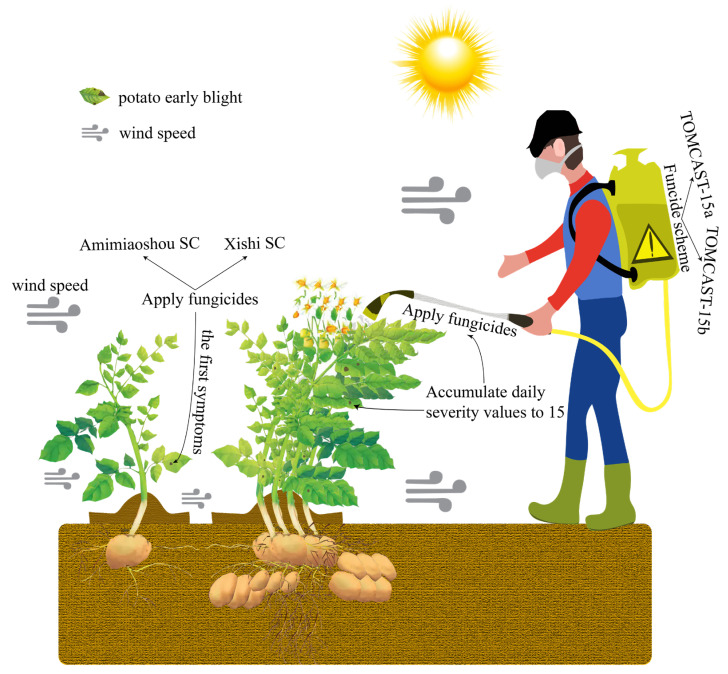
Flow chart of different application schemes for potato early blight.

The experiments were conducted in a randomized complete-block design with five treatments. Each treatment was replicated four times. The trial plots consisted of a 9 m × 3.75 m area with five ridges in a plot (Figure 5). The trial cultivar was Xueyu1, which was susceptible to potato early blight. The factor used in the experiments was fungicide application schedules. Detailed descriptions of the fungicide schedules are shown in Table 5. Amimiaoshou SC (20% azoxystrobin + 12.5% difenoconazole, Quadris Top 325SC, Syngenta Crop Protection, Inc., Basel, Switzerland) and Xishi SC (4% difenoconazole + 40% chlorothalonil, Syngenta Crop Protection, Inc., Basel, Switzerland) were used at a rate of 1 liter ha^−1^ to control early blight, according to the fungicide schedules.

### 4.2. Cultural Practices, Insect Control and Late Blight

The standard treatment in the experiments (for example, fertilizer application, insect and weed control, and irrigation) were carried out from 2020 to 2022, as shown in Table 6. To avoid the influence of late blight, ruifan SC (430 g·ha^−1^, 23.4% Mandipropamid, Syngenta Crop Protection, Inc., Basel, Switzerland) was applied to suppress late blight and ensure that early blight was the main potato disease. Mandipropamid did not have a special effect on early disease; therefore, it did not affect the experimental purpose of this study [17]. All pesticide applications were carried out with the “Guardian” brand backpack hand sprayer.

### 4.3. Weather Data Monitoring

Meteorological parameters (temperature, RH, and precipitation) were recorded by using a portable weather station placed in the study field positioned at a 1 m height. Leaf-wet hours were defined as hours with RH > 88% [10]. Using Python to rewrite the programming language of the previous R language to run the TOMCAST model required the use of hourly temperature and estimated leaf humidity data.

### 4.4. The TOMCAST Model Implementation

TOMCAST is a weather-based model [13] derived from the dew sub-model of the FAST model [14]. In this model, the DSV was used as a function of duration, leaf wetness (h), and the average temperature during the leaf wetness period to evaluate the suitability of early infestation on a given day in numerical form. The DSV ranged from 0 (no risk) to 4 (high risk) [13]. The model was originally used to predict tomato early blight, septoria leaf spot, and anthracnose [27]. In practice, the daily DSVs are added up until a threshold is reached (for example, 15), at which point fungicide treatment is recommended [28]. If the threshold for DSV accumulation is higher, then the time interval between subsequent applications is longer, and vice versa.

In this trial, the physiological days value was increased from the potato sowing stage [29,30] to 300, the calculation of the fungicide application threshold was started, and the TOMCAST threshold (15) was used for fungicide application. The TOMCAST threshold was selected based on previous studies in which the TOMCAST threshold (15) could achieve the best effect [10,12]. The TOMCAST model was originally designed to be located at the University of Pennsylvania [14]; therefore, we downloaded data from the nearest meteorological site at the Pennsylvania State University Plant Pathology farm in the pre-experimental phase of the study to compare with meteorological data from the Zhangbei site in the Bashang grassland of Hebei Province, China, in the same year and found that the average summer (May–September) temperatures at the two sites differed by approximately 4 °C. In addition, we analyzed the pre-experiment data in 2019 and found that the lowest temperature with a relative humidity greater than 88% at the test site was 7.02 °C, as required in the TOMCAST model for average temperature, hours with RH greater than 88%, RH, and total rainfall data. Previous studies reported that when the temperature was below 7 °C or above 25 °C, *Alternaria* conidia is significantly reduced [31,32]. Therefore, a lower temperature is not conducive to the germination and flow of *Alternaria* conidia in potato early blight. As a result, we changed the minimum temperature from 13 °C to 7 °C in the TOMCAST model. The interval between fungicide applications can be calculated using the daily disease severity values accumulated at 15; however, if there has been less than seven days since the last application, no fungicide was used.

### 4.5. Disease Assessment

Early blight assessments were performed starting from the onset of symptoms, or rating began after observation of 1% disease severity. Disease severity was evaluated with the percentage of diseased leaves and disease index on a plot basis, according to the disease leaf grading scale in the Pesticide Field Efficacy Test Criteria. Disease severity was rated every seven days in the center of four rows in each plot until one week before harvest but not for the plants at each end of each row.

The AUDPC was then calculated from the disease assessment data according to the mid-point method [33] to express the cumulative incidence of leaf and infection occurring, with the following formula:

(1) $$\begin{array}{c} AUDPC=\sum_{i=1}^{n}\left[\frac{\left(x_{i+1}+x_{i}\right)}{2}\times \left(y_{i+1}-y_{i}\right)\right] \end{array}$$

where $x_{i}$ = disease severity (per unit) at the ith observation, $y_{i}$= time (days) at the ith observation, and n = the total number of observations.

### 4.6. Yield and Quality Estimates, and Statistical Analyses

When the potato growing season ended, potato tubers were harvested from the middle two rows of each plot. The tubers were dug out with a tractor-mounted harvester fitted with a weighing scale to measure the tuber weight as soon as the potatoes were removed from the soil. Tubers (5 kg) were randomly taken from harvested potatoes, and the dry matter content of potato pieces was calculated using the water-specific gravity method [34]. The reducing sugar, starch, and protein contents of potato tubers were determined using the 3,5-dinitrosalicylic acid colorimetry [35], iodine colorimetry [36], and Kjeldahl [37] methods, respectively.

An analysis of variance (ANOVA) was used to test for differences between the five treatments from 2020 to 2022; Fisher’s least significance differences (LSD) were calculated at the 5% level of significance, evaluating the performance of the treatments on the measured variables. Each experiment was repeated five times, and the experimental data are expressed as mean ± standard deviation (mean ± SD).

## 5. Conclusions

This study demonstrated that the TOMCAST 15 DSV model could better suppress early blight in potatoes by applying fungicides less frequently and without any yield loss. In addition, this study also indicated that the TOMCAST 15 DSV model, combined with Amimiaoshou SC fungicide, could achieve the same effect as the standardized treatments (Amimiaoshou SC and Xishi SC) in increasing the dry matter, starch, protein, and reducing sugar contents of potatoes. Therefore, TOMCAST-15 × Amimiaoshou SC was a well-evaluated model in this study. This method can be considered as an alternative to both Amimiaoshou SC and Xishi SC application. The results of this study confirmed the applicability of the TOMCAST model in China. However, selecting the optimal thresholds for the TOMCAST model in Chinese potato growing areas needs to be further explored.

## Figures and Tables

**Table 1:** Dates of spraying fungicide to control potato early blight during the experiment in 2020.

Date ^1^	4 July	11 July	18 July	25 July	1 August	8 August	15 August	22 August
Fungicide schedule								
Untreated	−	−	−	−	−	−	−	−
Amimiaoshou SC			+	+	+	+	+	+
Xishi SC			+	+	+	+	+	+
TOMCAST-15a		+		+		+		+
TOMCAST-15b		+		+		+		+

^1^ The date fungicide was sprayed is represented by the plus sign (+), and the minus sign (−) represents a day on which no fungicide was sprayed for potato early blight.

**Table 2:** Dates of spraying fungicide to control potato early blight during the experiment in 2021.

Date ^1^	7 July	14 July	21 July	28 July	4 August	11 August	18 August	25 August
Fungicide schedule								
Untreated	−	−	−	−	−	−	−	−
Amimiaoshou SC			+	+	+	+	+	+
Xishi SC			+	+	+	+	+	+
TOMCAST-15a	+		+		+		+	
TOMCAST-15b	+		+		+		+	

^1^ The date fungicide was sprayed is represented by the plus sign (+), and the minus sign (−) represents a day on which no fungicide was sprayed for potato early blight.

**Table 3:** Dates of spraying fungicide to control potato early blight during the experiment in 2022.

Date ^1^	10 July	17 July	24 July	31 July	6 August	13 August	20 August	27 August
Fungicide schedule								
Untreated	−	−	−	−	−	−	−	−
Amimiaoshou SC				+	+	+	+	+
Xishi SC				+	+	+	+	+
TOMCAST-15a	+			+			+	
TOMCAST-15b	+			+			+	

^1^ The date fungicide was sprayed is represented by the plus sign (+), and the minus sign (−) represents a day on which no fungicide was sprayed for potato early blight.

**Table 4:** Mean tuber yield of different fungicide schedules from 2020 to 2022.

Fungicide Schedule	Tuber Yield (Tons ha^−1^) *		
	2020	2021	2022
Amimiaoshou SC	39.36 ± 1.46	36.78 ± 1.31	36.78 ± 1.46
Xishi SC	37.53 ± 1.45	34.66 ± 2.25	36.46 ± 1.70
TOMCAST-15a	36.00 ± 1.12	33.21 ± 3.18	36.50 ± 2.32
TOMCAST-15b	35.20 ± 1.42	32.52 ± 2.82	35.21 ± 1.91
Untreated	33.35 ± 0.66	29.96 ± 1.31	29.96 ± 0.91

* Tuber yields in the table are mean values of five replicates and are expressed with their standard errors. Followed by the same letter indicates no significant difference and vice versa.

**Table 5:** Description of the fungicide schedule used to control early blight during the field trial from 2020 to 2022.

Fungicide Schedule	Description
Untreated	No fungicide application to control early blight. The untreated served as a reference for the general development of early blight.
Amimiaoshou SC	Fungicide application started when the first disease symptoms appear on leaves in the trial and continued at a 7-day interval.
Xishi SC	Fungicide application started when the first disease symptoms appear on leaves in the trial and continued at a 7-day interval.
TOMCAST-15 a	Beginning with the accumulation of P-days to 300 days, the Amimiaoshou SC fungicide application was started at each accumulation of DSV to 15.
TOMCAST-15 b	Beginning with the accumulation of P-days to 300 days, the Xishi SC fungicide application was started at each accumulation of DSV to 15.

a Amimiaoshou SC (20% azoxystrobin+12.5% difenoconazole) at a rate of 1 liter ha^−1^ was used to control early blight. b Xishi SC (4% difenoconazole + 40% chlorothalonil) at a rate of 1 liter ha^−1^ was used to control early blight.

**Table 6:** Field operations and agronomic practices undertaken from 2020 to 2022.

Activity	2020	2021	2022
Planting	2 May	5 May	7 May
Date of 50% emergence	6 June	10 June	12 June
Fertilizer application	30 April	2 May	31 April
Rate of fertilization	900 kghm^−2^12-18-15 N-P-K	900 kghm^−2^12-18-15 N-P-K	900 kghm^−2^12-18-15 N-P-K
Irrigation	35 mm 20 June, 10 and 28 July, 11 August	35 mm 25 June, 16 and 29 July, 7 August	35 mm 28 June, 7, 15, 23 and 30 July, 18 August
Insect control ^a^	19 June and 25 July	24 June and 28 July	26 June and 28 July
Weed control ^b^	30 May and 26 June	30 May and 26 June	30 May and 26 June

^a^ (1) Ai meile WDG (120 g·ha^−1^,70% imidacloprid, Bayer Crop Science (China) Co., Ltd., Zhejiang, China), (2) Te fuli SC (120 g·ha^−1^, 22% sulfoxaflor, Kedihua Agricultural Science and Technology Co. Ltd., Beijing, China). ^b^ (1) Jin Duer SC (100 g·ha^−1^, 96% S-metolachlor, Syngenta Crop Protection, Inc., Basel, Switzerland). (2) Dao sida WP (150 g·ha^−1^, 80% oxadiargyl, Bayer Crop Science Co., Ltd., Zhejiang, China).

## Data Availability

Not applicable.
